# Pediatric discordant lymphoma with classic Hodgkin lymphoma in cervical lymph nodes and high-grade B-cell non-Hodgkin lymphoma in bone marrow: a case report from Pakistan

**DOI:** 10.3332/ecancer.2024.1702

**Published:** 2024-05-10

**Authors:** Namrita Rai, Zeenat Amna Azhar, Neelum Tahir Kheli, Fozia Lateef, Omer Javed, Muhammad Rafie Raza

**Affiliations:** 1 Department of Pediatric Hematology and Oncology, Indus Hospital & Health Network, Karachi 75190, Pakistan; 2 Department of Hematology, Indus Hospital & Health Network, Karachi 75190, Pakistan; 3 Department of Histopathology, Indus Hospital & Health Network, Karachi 75190, Pakistan; a https://orcid.org/0000-0002-6300-8961

**Keywords:** discordant, lymphoma, Hodgkin, pediatric, non-Hodgkin, cancer, child, Pakistan

## Abstract

Discordant lymphoma (DL) is an uncommon condition in which two or more histologically different types of lymphomas are present at distinct anatomical sites in the same patient. Here, we report a case of a pediatric patient under 10 years old presenting with symptoms of general sickness with cervical lymphadenopathy, abdominal distension and an abdominal mass. Upon conducting investigations, classic Hodgkin lymphoma (CHL) was detected in the cervical lymph nodes, and high-grade B-cell non-Hodgkin lymphoma was detected in the bone marrow and abdominal mass. The patient was therefore diagnosed with DL. The boy was initially diagnosed with CHL but proceeded to have aggressive disease progression, due to which further workup was done. In the past, literature reports have been published for adult cases of DL, and currently, research is being conducted to formulate treatment protocols for it. However pediatric cases of DL remain widely undiscussed. Since we are dealing with a rare or widely underreported condition, we found it significant to elaborate on its clinical presentation, treatment plan, complications and prognosis.

## Background

Discordant lymphoma (DL) refers to the presence of multiple histologic subtypes of lymphoma in the same patient at two separate anatomic sites [1]. The prevalence of DL has not accurately been quantified in large population samples. While it is considered to be a rare disorder in some recent literature [3], it has also been said that the coexistence of primary and indolent lymphomas may not be as rare as previously assumed [4]. Classic Hodgkin lymphoma (CHL) and non-Hodgkins lymphoma (NHL) were previously assumed to be unrelated; however, it has now been established that the different types of cell lines originated from a common germinal-center B-cell precursor [5]. This may explain subsequent developments of NHL to CHL in patients [6] but both diseases coexisting at the same time in different anatomical locations (discordant morphology) is still an interesting and unexplained phenomenon with unestablished treatment guidelines. There have recently been increased attempts at discussing DL in adults [7, 8] however, there is little to no literature on its presentation and treatment guidelines in children. We report here a discordant case of CHL and high-grade B-cell non-Hodgkin lymphoma (B-Cell NHL) in a 6-year-old boy presenting to the Pediatric Hematology and Oncology department in Karachi, Pakistan.

## Clinical presentation and case details

A 6-year-old male resident of Sukkur, Sindh, Pakistan, presented to our Emergency Room in Karachi with complaints of fever, abdominal distension and neck swelling for the past 1 month. He also reported bone pain and generalised weakness. The patient had a family history of tuberculosis (multiple aunts and uncles). He had no history of prior blood transfusions and no significant past medical or surgical history.

On examination, the patient was alert and oriented to his surroundings. He appeared thin, lean and malnourished with height and weight less than 3rd centile for his age. He was positive for bilateral cervical lymphadenopathy in the neck. The patient’s abdomen appeared distended, and a firm abdominal mass was palpable in the umbilical region.

Initial workup showed a complete blood count report consisting of Hb 10.5 g/dL, MCV 57.1 fL, TLC of 7.8 × 10^9^/L and platelets 547 × 10^9^/L. Lactate dehydrogenase (LDH) report showed 951 U/L (normal range: 125–220). Ultrasound Abdomen showed moderate ascites with splenic deposits. computed tomography (CT) scan was consistent with huge deposits encasing the small and large bowel loops. One of the deposits causing compressive effects on major blood vessels measured 7.0 × 4.5 cm in transverse (TR) and antero-posterior (AP) dimensions. Another deposit in the paraaortic region measured 2.4 × 3.9 cm in AP and TR dimensions. Small and large bowel loops showed thickening with dilatation and thickening of the terminal ileum making a sandwich sign. Differential diagnoses for the patient included tuberculosis and lymphoma.

A cervical lymph biopsy node was conducted, which concluded a diagnosis of CHL. The patient underwent a positive emission tomography and computed topography scan and bilateral bone marrow aspirate and trephine biopsy for further staging. Meanwhile, the first cycle of cyclophosphamide, vincristine sulfate, dacarbazine and prednisone was given for Hodgkins lymphoma as per the national protocol of the Pakistan Society of Pediatric Oncology (PSPO) awaiting bone marrow trephine reports. Bone marrow biopsy findings were consistent with high-grade B-Cell NHL.

Due to the inconsistent results of the biopsy reports in different anatomical sites, an excisional cervical lymph node biopsy was conducted to reevaluate the cervical lymph node. The second cervical lymph node biopsy concluded with the same results as that of the first – also consistent with CHL (Nodular Sclerosing subtype). All necessary clerical checks were performed to confirm that the correct biopsy specimen was sent and analysed for both bone marrow and cervical lymph node

Over the course of treatment, the patient developed fever, ascites and unilateral facial nerve palsy, due to which he was admitted. A CT scan abdomen with contrast was repeated showing the development of a large right paracardiac lesion measuring 3.0 × 4.7 cm. There was interval development of multiple pancreatic lesions, one of them measuring 4.0 × 2.5 cm. However, a previously noted large insinuating mass lesion within the mesentery had reduced in size from 7.5 × 4.5 cm to 4.5 × 3.5 cm in the current scan.

An ultrasound biopsy of the patient’s abdominal mass was performed which was consistent with high-grade B-cell NHL, with subtype differential diagnoses Burkitt lymphoma and diffuse large B-cell lymphoma. Cerebrospinal fluid (CSF) cytology was conducted which showed lymphoma cells signifying central nervous system (CNS) disease involvement.

Based on biopsy findings, the case was concluded to be ‘DL’ with CHL in the cervical lymph nodes and NHL in the bone marrow, abdominal mass and CSF.

After conducting a multidisciplinary tumour board meeting and extensive literature search, it was decided that treatment and workup would be conducted according to the more aggressive disease classification, which was B-cell NHL. Treatment with rituximab, cyclophosphamide, vincristine, prednisone, doxorubicin and methotrexate (R-COPADM) was begun as per the national protocol of PSPO.

The patient developed carbapenem-resistant enterobacteriaceae (CRE) sepsis during his hospital stay and was shifted to the pediatric intensive care unit. His liver function tests became deranged. The patient eventually developed pleural effusion. He was kept on high-flow oxygen therapy but unfortunately developed multi-organ failure and was not able to survive due to the complications of CRE sepsis.

### Histopathology

**Figure 1.**
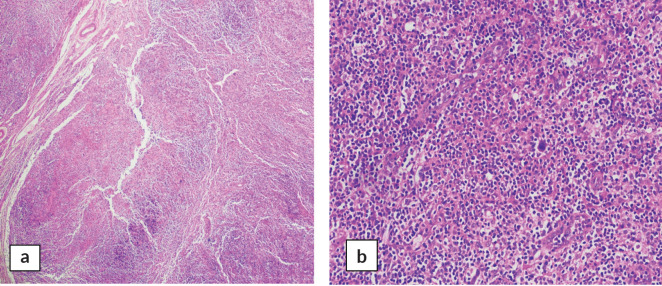
Sections of cervical lymph node biopsy showing infiltration with Reed-Sternberg cells in a reactive background of lymph node tissue. (a): H&E 4× and (b): H&E 20×.

**Figure 2.**
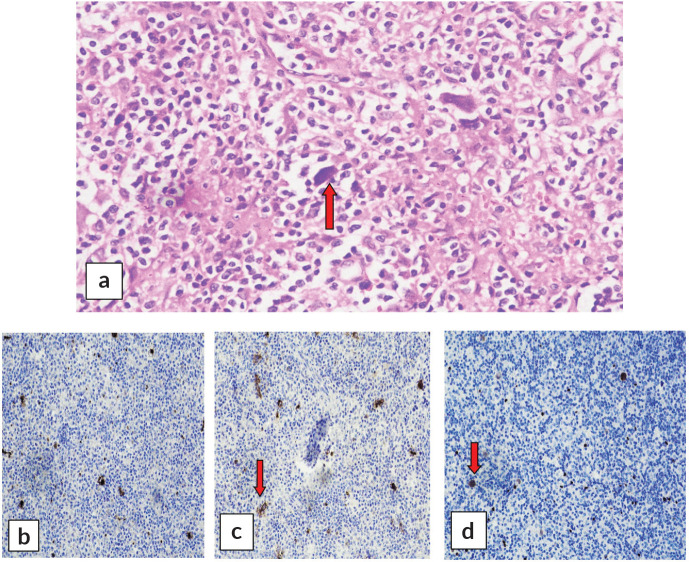
(a): Reed-Sternberg cells (red arrow) at 40×. Immunohistochemical stains showing positivity of these cells for (b): CD15, (c): CD30 and (d): PAX-5.

A cervical lymph node biopsy was performed with the clinical suspicion of lymphoma (Figure 1). Histopathological examination of the lymph node was done and revealed complete effacement of nodal architecture by scattered large atypical cells with intervening thin fibrous septae imparting a nodal architecture. The large atypical cells had moderate eosinophilic cytoplasm. Nuclei were vesicular with prominent eosinophilic nucleoli. Occasional binucleated and multinucleated cells were also noted. The background showed lymphocytes, histiocytes, few plasma cells and eosinophils. The following panel of immunohistochemical markers was performed and the results were as follows: the large cells were CD30 and CD15 positive, and negative for CD45 (Figure 2). PAX-5 was dim positive in large atypical cells. CD20 was negative in large atypical cells and positive in reactive B-lymphocytes in the background. CD3 negative in large atypical cells, and positive in background T-lymphocytes. A diagnosis of CHL was made.

Bone marrow biopsy was performed for the staging of the disease, i.e., Hodgkin lymphoma.

**Figure 3.**
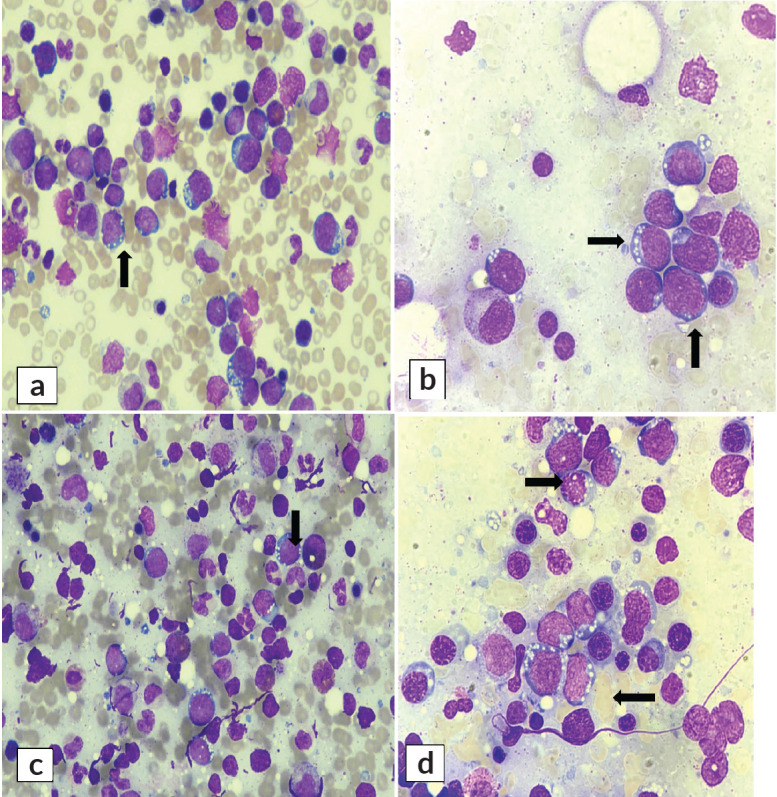
Right-sided bone marrow aspirate at (a): ×40 and (b): ×100. Left-sided bone marrow aspirate at (c): ×40 and (d): ×100. Aspirate exhibits mononuclear malignant cells having deeply basophilic cytoplasm with prominent vacuolation (Wright Giemsa stain).

The bone marrow aspirate smears unexpectedly revealed infiltration by mononuclear cells (Figure 3). These were medium to large in size, characterised by a high nuclear-to-cytoplasmic ratio, coarse nuclear chromatin, indistinct nucleoli and moderate amount of deep basophilic agranular cytoplasm with multiple fine vacuolations. The overall morphology of cells was consistent with high-grade non-Hodgkin large-cell lymphoma. Residual cells showed a spectrum of hematolymphoid cells exhibiting trilineage hematopoiesis with adequate megakaryocytes.

**Figure 4.**
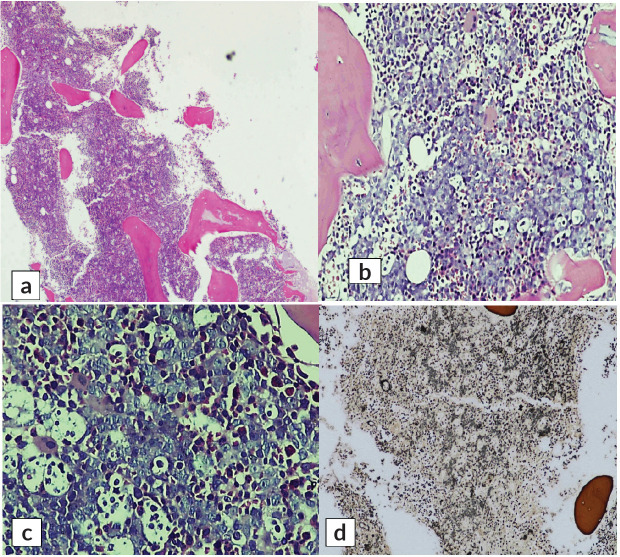
H&E stained sections of right-sided bone marrow trephine biopsy exhibiting infiltration by lymphoma cells giving rise to starry sky appearance. (a): ×10, (b): ×20, (c): ×40 and (d): reticulin stain showing MF-1.

**Figure 5.**
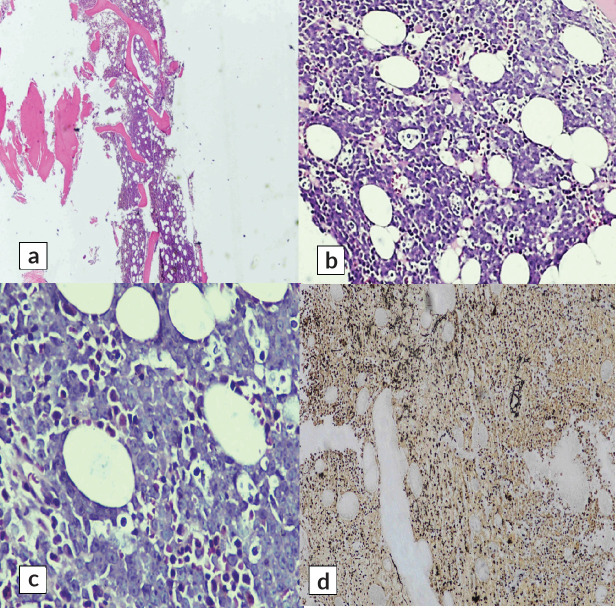
H&E stained sections left-sided trephine biopsy exhibiting infiltration by lymphoma cells. (a): ×10, (b): ×20, (c): ×40 and (d): reticulin stain showing MF-0.

**Figure 6.**
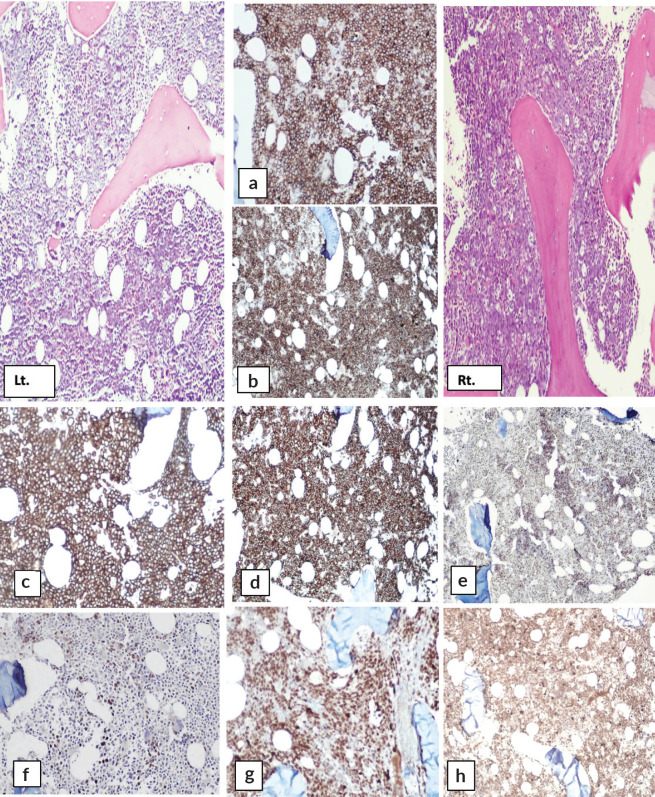
Immunohistochemical stains on bone marrow biopsy showing positivity for: (a&b): CD10, (c&d): CD20, (e&f): BCL-6 and (g&h): Ki-67.

H and E-stained section of bilateral bone marrow trephine biopsy (Figures 4 and 5) revealed adequate length trephine cores with overall cellularity of 90%–95% exhibiting areas of infiltration by mononuclear lymphoma cells in sheets, having morphology like that of aspirate. Tingible body macrophages are also seen in the involved areas, giving a starry sky appearance. Residual hematopoiesis was suppressed. Mild background fibrosis was also noted in parts of trephine upon Reticulin staining. By immunohistochemical stains, the neoplastic cells were positive for CD20, CD10, BCL-6 and dim positive for c-MYC (Figure 6). Ki-67 proliferation index was 70%–75% of neoplastic cells. The abnormal infiltrate was negative for CD3, CD30 and BCL-2. Overall morphological and immunohistochemical findings were suggestive of high-grade B-Cell NHL.

The immunohistochemical findings are shown in Figure 6.

This was an interesting case – and very rare in the region – where cervical lymph node biopsy of the patient was consistent with CHL, while the findings in bone marrow biopsy were consistent with B-Cell NHL. All the checks were performed and slides staining along with immunohistochemical markers had been repeated and rechecked. After discussing the case in a tumour board meeting, a decision was made to perform an abdominal mass biopsy.

**Figure 7.**
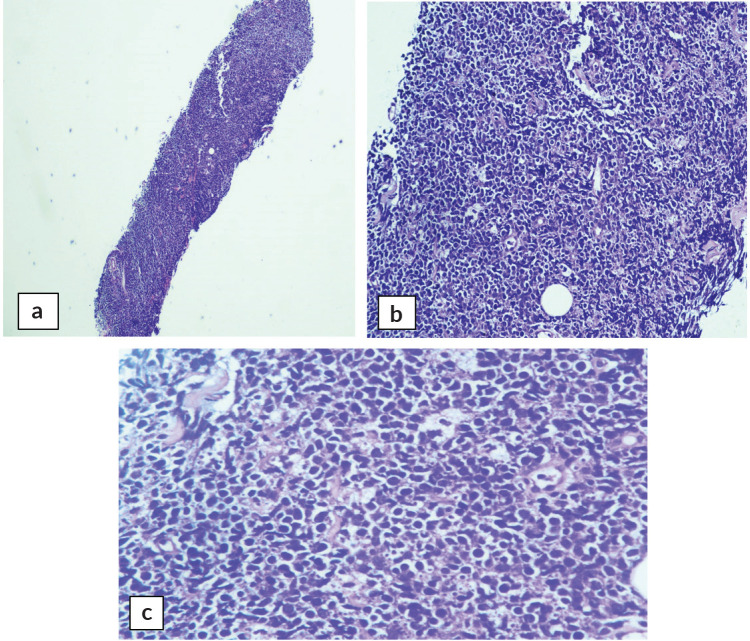
Abdominal mass core biopsy showing infiltration with sheets of medium and large cells and karyorrhectic debris. (a): 4×, (b): 20× and (c): 40×.

**Figure 8.**
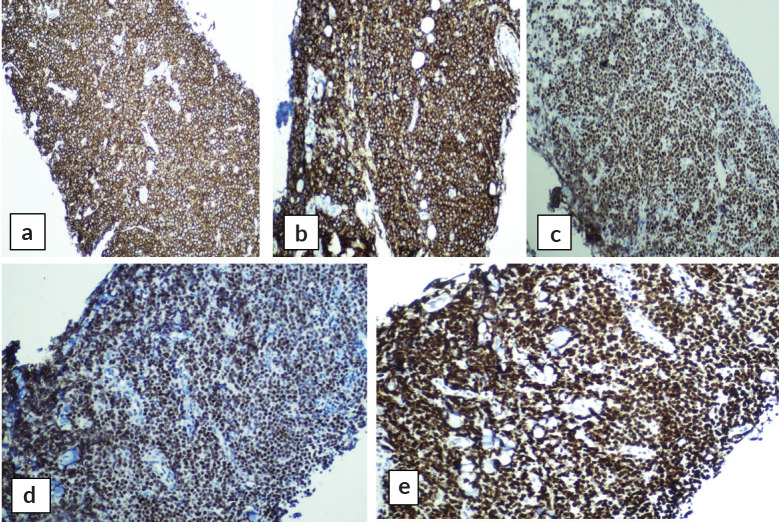
Immunohistochemical stains on abdominal mass biopsy showing positivity for (a): CD20, (b): CD10, (c): BCL-6, (d): C-myc and (e): Ki-67.

Abdominal mass biopsy (Figure 7), showed sheets of intermediate to large-sized cells. The cells have enlarged, hyperchromatic nuclei with irregular nuclear contours and prominent nucleoli. Numerous apoptotic bodies and mitotic figures are seen. By immunohistochemical stains, the neoplastic cells were positive for CD10, CD20, BCL-6, C-myc and Ki-67 proliferation index was found to be positive in up to 95% of the cells (Figure 8).

## Discussion

The patient, although diagnosed with nodular sclerosing CHL in a cervical lymph node, continued to exhibit an unusual prognosis that led to a high index of suspicion for a more aggressive subtype of lymphoma. This was due to the presence of an abdominal mass, along with symptoms of sickness such as abdominal pain and fever spikes. The patient continued to develop complications including facial nerve palsy, ascites and pleural effusion, which further strengthened this suspicion.

A bone marrow biopsy was conducted since it holds high diagnostic value and can shed light on clinical outcomes and impact treatment selection [9]. However, the bone marrow biopsy along with the biopsy of the abdominal mass histologically showed the presence of NHL. After necessary clerical checks and repeat biopsy, the case was diagnosed as DL due to the presence of different histological subtypes of lymphoma in different anatomical sites in the patient. Flow cytometry and molecular diagnostics were not performed due to the unavailability of these facilities at the center.

While clinical presentations of DL have been discussed in the past [1], these are either primarily inclusive of adults or clump pediatric cases with adults. This leads to the existence of limitations for clinicians and pathologists when it comes to the diagnosis of DL in children. Large-scale pediatric studies need to be conducted to enable pediatric oncologists to aptly identify, approach and treat DL in a timely fashion.

Furthermore, the lack of relevant literature leads to a gap in therapeutic guidelines. Some studies have recently been conducted on adult patients to evaluate the impact of treatment regimens aimed at DL, namely Park *et al* [2] showing discordant bone marrow involvement leading to a negative prognostic impact on progression-free survival and overall survival of patients receiving R-CHOP therapy [2]. No such studies evaluating treatment regimens have been conducted in the pediatric population. According to previous studies, the established reasonable approach to management is to treat the more aggressive subtype of lymphoma first [10, 11]. After conducting a multidisciplinary tumour board meeting, the patient was therefore given R-COPADM to treat NHL, as he had stage IV disease with CNS and bone marrow involvement. However, due to complications of neutropenia and CRE sepsis, the patient was unable to survive.

We believe detailed diagnostic and therapeutic guidelines for pediatric DL cases would reduce the time to establish a diagnosis and, therefore, reduce the time taken to administer effective treatment for the patient. Establishing standardised treatment guidelines for pediatric lymphoma patients is especially important since according to recent literature, a discordant diagnosis was found to be associated with an approximate three-fold increased risk of death [12].

## Conflicts of interest

No potential conflict of interest exists for any of the authors involved.

## Funding

No specific funding and/or grant was received for the purpose of this research.
